# Microglia in the spinal cord stem cell niche regulate neural precursor cell proliferation via soluble CD40 in response to myelin basic protein

**DOI:** 10.1093/stmcls/sxae076

**Published:** 2024-11-16

**Authors:** Nishanth Lakshman, Filip Stojic, Cindi M Morshead

**Affiliations:** Department of Surgery, Division of Anatomy, University of Toronto, Toronto, ON M5S 1A8, Canada; Institute of Medical Sciences, University of Toronto, Toronto, ON M5S 1A8, Canada; Institute of Medical Sciences, University of Toronto, Toronto, ON M5S 1A8, Canada; Department of Surgery, Division of Anatomy, University of Toronto, Toronto, ON M5S 1A8, Canada; Institute of Medical Sciences, University of Toronto, Toronto, ON M5S 1A8, Canada; Institute Biomedical Engineering, University of Toronto, Toronto, ON M5S 3G9, Canada; Donnelly Centre for Cellular and Biomolecular Research, University of Toronto, Toronto, ON M5S 3E1, Canada

**Keywords:** neural stem cells, myelin basic protein, spinal cord, microglia, stem cell niche

## Abstract

Neural stem cells (NSCs) are found along the neuraxis of the developing and mature central nervous system. They are found in defined niches that have been shown to regulate NSC behavior in a regionally distinct manner. Specifically, previous research has shown that myelin basic protein (MBP), when presented in the spinal cord niche, inhibits NSC proliferation and oligodendrogenesis. Herein, we investigate the cell-based mechanism(s) underlying this spinal-cord niche-derived MBP-mediated inhibition. We used reporter mice to sort for subpopulations of cells and found that spinal cord niche-derived microglia release a soluble factor in response to MBP that is responsible for NSC inhibition. Microglia, but not other niche cells, release soluble CD40/TNFRSF5 (sCD40) in the presence of MBP which may indirectly reduce activation of transmembrane CD40/TNFRSF5 receptor on both spinal cord and brain NSCs. This is consistent with sCD40 binding to CD40 ligand (CD40L) thereby preventing CD40 receptor binding on NSCs and inhibiting NSC proliferation. The identification of the cell-based mechanism that regulates NSC behavior in response to MBP, which is dysregulated in injury/disease, provides insight into a potential target for strategies to enhance neural repair through endogenous stem cell activation.

## Graphical Abstract

**Graphical Abstract:**
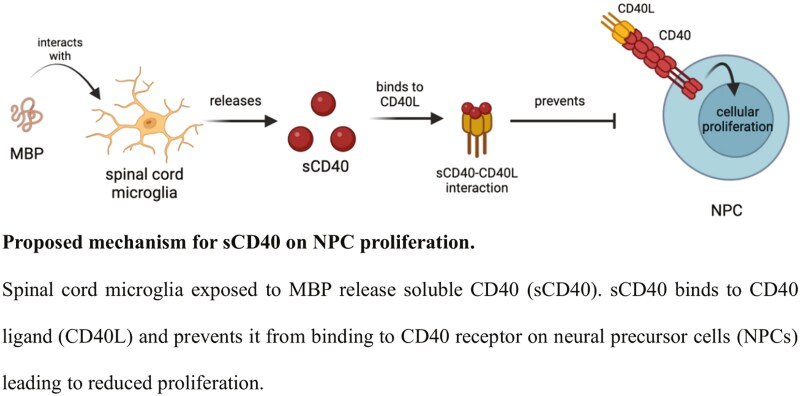

**Significance statement**

Here, we demonstrate that microglia isolated from the spinal cord niche respond to myelin basic protein by releasing soluble CD40 which inhibits neurosphere formation. These findings identify cellular and molecular pathways that regulate NSC behavior which can be targeted to enhance stem-cell based neural repair.

## Introduction

The adult mammalian central nervous system (CNS) has limited capacity for self-repair following injury. The discovery of neural stem cells (NSCs) in specialized niches in the mammalian brain and spinal cord afforded the promise of enhanced regenerative capacity through the manipulation of NSCs and their progeny (neural precursor cells, NPCs). Recent work has shown that activating endogenous NPCs in the spinal cord using drugs such as metformin, is correlated with functional improvements following SCI.^1,2^

The NSC niche is composed of neighboring cells that provide cues through cell-cell contact and secreted factors^3,4^ that regulate NSC behavior. These niche cells include astrocytes, microglia, ependymal cells, endothelial cells, oligodendrocytes, tanycytes, and CSF-contacting neurons.^5-7^ Under homeostatic conditions, a number of factors have been shown to regulate NSC kinetics including growth factors, cytokines, and small molecules released from neighboring cells.^8-10^ Following injury, microglia and astrocytes play a prominent role in NSC activation and regulation through the release of factors into the injured NSC environment.^11,12^ CD40 (also known as TNFRSF5) is a member of the tumor necrosis factor receptor superfamily and is known to play a role in microglia, B cell, and cancer cell proliferation.^13-15^ CD40 is a transmembrane protein that acts as a receptor for CD40 ligand (CD40L). Binding of CD40 to CD40L induces proliferation.^15^ Soluble CD40 (sCD40) can be produced by enzymatic cleavage of the extracellular domain or by expression of the sCD40 mRNA isoform.^16^ sCD40 can bind and sequester CD40L, thus preventing binding to the membrane-bound receptor and inhibiting proliferation.^17^ The characterization of factors that regulate NPCs in response to injury has implications for neural repair.

One factor that negatively regulates NSC proliferation and oligodendrogenesis, is myelin basic protein (MBP).^18^ MBP is a prominent protein in mature myelin that is released into the microenvironment following injury/disease.^19-21^ Most interestingly, the inhibitory effects of MBP are only observed when MBP interacts with cells derived from the spinal cord NSC niche—the periventricular zone (PVZ) lining the central canal. MBP does not lead to the release of an inhibitory factor if presented in the brain NSC niche; the subventricular zone (SVZ) lining the forebrain lateral ventricles.^7,22^ However, forebrain NSCs are still responsive to the inhibitory factor released from the spinal cord niche. The cell-based mechanism underlying the effects of MBP on NSC behavior has not been established.

Herein we isolate distinct populations of niche cells and identify PVZ microglia as the cell that responds to MBP and releases a soluble factor that inhibits NSC proliferation. We used mass fractionation, cytokine arrays, and ELISA to identify sCD40 as the inhibitory factor released in response to MBP. This mechanism has implications for NSC-based neural repair.

## Materials and methods

### Mice

Mice were housed within the Department of Comparative Medicine at the University of Toronto. Experiments were conducted following the approval of the Animal Care Committee and in accordance with the Guide to the Care and Use of Experimental Animals provided by the Canadian Council of Animal Care. C57/Bl6 mice (Charles River), CX3CR1-GFP mice (B6.129P2(Cg)-*Cx3cr1*^*tm1Litt*^/J*CX3CR1*-GFP) (https://www.jax.org/strain/005582) and GFAP-GFP mice (FVB/N-Tg(GFAPGFP)14Mes/J) (https://www.jax.org/strain/003257) were bred in house and used at 6-8 weeks of age.

### Neurosphere cultures

The protocol for isolation and culturing of NSCs and their respective niches has been previously described for the brain^23^ and spinal cord^24^ and was slightly modified herein. Briefly, mice were euthanized and the spinal cord and/or forebrain were removed. The PVZ of the spinal cord or brain SVZ was dissected and dissociated into single cells using enzymatic treatment (1.33 mg/mL trypsin and 0.83 mg/mL hyaluronidase; T1005-1G/ H6254-1G, Sigma, Canada) and mechanical trituration. Dissections included the PVZ and surrounding gray matter (70 µm from the lumen of the central canal). Cells were plated at 10 cells/μL in serum-free media (Neurobasal-A medium (10888022, Invitrogen, Canada) containing L-glutamine (2 mM, 25030164, Invitrogen), penicillin/streptavidin (100 U/0.1 mg/mL, 15140163, Invitrogen)) with mitogens (epidermal growth factor [20 ng/mL]; fibroblast growth factor [10 ng/mL]; heparin [2 μg/mL], Sigma, referred to as EFH). The numbers of neurospheres were counted at 7 days in vitro. Secondary neurospheres were derived from passaged primary neurospheres in the same media conditions.

### Neurosphere culture with myelin depletion

Myelin was removed from primary cultures using an established Percoll protocol previously used by our group and others.^22,25^ Percoll Plus solution (23%, Millipore-Sigma, GE17-5445-01) suspended in 1× sterile phosphate-buffered saline (PBS) was added to enzymatically digested tissue and triturated. The solution was centrifuged for 15 minutes at 600 g. Myelin supernatant was removed, and cells were washed with serum-free media, triturated, and centrifuged again at 400 g for 3 min. Supernatant was removed and cells were plated in EFH.

### Conditioned media

To generate conditioned media (CM), serum-free media used for the neurosphere assay was added to cell cultures plated at 50 cells/µL for 48 hours. The CM was then collected and filtered through a 0.22-μm syringe filter unit (Millipore, Toronto, Canada, http://www.cedarlanelabs.com/). CM was then plated onto cells at clonal density (10 cells/µL) to grow neurospheres for 7 days.

### Mass fractionation

Mass fractionation of spinal cord CM (spCM) was performed using Amicon Ultra-14 centrifugal filter units of 5 different sizes—3kDa, 10kDa, 30kDa, 50kDa, and 100kDa. Serial filtration was performed from the 100 kDa filter unit (100 kDa) using smaller molecular weight filter units. spCM was separated into 5 molecular weight fractions (3-10 kDa, >10-30 kDa, >30-50 kDa, >50-100 kDa, and >100 kDa) from cells grown in the presence or absence of MBP, as described.

### Cell sorting

Fluorescence-activated cell sorting (FACS) was performed for microglia and astrocytes using a modified protocol.^18^ PVZ tissue of CX3CR1-GFP mice and GFAP-GFP mice was pooled from 2 mice per group for each FACS experiment. Negative controls were age-matched wild-type (WT) C57Bl/6 mice. Positive controls were obtained from CX3CR1-GFP or GFAP-GFP brains. Tissue was prepared by mincing in artificial cerebrospinal fluid [6.2 mL 2M NaCl (Bioshop, SOD002), 0.5 mL 1M KCl (Sigma, P5405), 0.13 mL MgCl_2_•6H_2_O (Sigma, M2393), 16.9 mL 155mM NaHCO_3_ (Sigma, S5761), 1 mL 1M glucose (Sigma, G6152), 1.852 mL 108 mM CaCl_2_, (Sigma, C7902), 1 mL penicillin-streptomycin (Wisent, 450-201-EL) in 72.4 mL ddH_2_O] and dissociated with the Papain Dissociation System (Worthington, LK003150) according to manufacturer’s instructions. Pellets were resuspended in ice-cold 23% Percoll Plus (Sigma-Aldrich, ON, Canada, GE17-5445-02). Samples were centrifuged for 15 minutes at 600 g at 4°C. Myelin residuals were removed from the Percoll gradient with a P1000 pipette. Samples were washed in phenol-free serum-free media and centrifuged for 5 minutes at 400 g at 4 °C, then resuspended in 600 μL of phenol-free serum-free media. Gating controls were resuspended in 200 μL phenol-free serum-free media. To identify dead cells, 4ʹ,6-diamidino-2-phenylindole (DAPI; Invitrogen, ON, Canada, D1306) (1:10,000) was added to the samples prior to straining through sterile 40 μm nylon cell strainers (Thermo Fisher Scientific, 22-363-547) into Falcon 5 mL round-bottom polypropylene tubes (Corning Life Sciences). Samples were kept on ice prior to sorting.

Cell sorting was performed using a BD FACS Aria IIIu (BD Biosciences) with a sheath pressure of 20 psi and 100 μm nozzle aperture. BD FACS Diva 8.0 software (BD Biosciences) was used for data acquisition and data was analyzed with FlowJo 9.3 (FlowJo LLC). Gating was performed using forward scatter area, side scatter area, and forward scatter height to exclude debris and doublets. Cells were collected into polypropylene tubes containing serum-free media on ice. Cells were transferred to 15 mL falcon tubes, centrifuged for 5 minutes at 400 g at room temperature then resuspended in serum-free media with EFH and plated at 50 cells/µL for CM production in the presence or absence of 50 μg/mL of MBP (UTC1—a gift from Dr. G Harauz (Guelph University))^22^ into 6-well Nunc polystyrene plates (Thermo Fisher Scientific).

### Cytokine array

The Proteome Profiler kit from R&D Systems (Proteome Profiler Mouse XL Cytokine Array, R&D Systems, ARY028) was used for the analysis of microglia derived spCM, with and without exogenously added recombinant MBP.^18^ Processing was performed as per the manufacturer’s instructions for cell culture supernatant. Image capture of membranes was performed using ChemiDoc XRS+ System (Bio-Rad Laboratories). Membrane blot intensity analyses were performed using ImageJ software (https://imagej.net/software/imagej/).

### Cytokine dose response

Cytokine dose-response curves were produced using recombinant forms of CD40 (R&D Systems, Catalog #: 1215-CD) and mResistin (R&D Systems, Catalog #: 1069-RN-050). Proteins were dissolved in sterile 1× PBS to make stock aliquots and mixed with serum-free media to attain a concentration of 4000 pg/mL. Serial dilution was performed using EFH media (2000, 1000, 500, 250, 125, and 62.5 pg/mL final concentrations). Passaged NPCs were plated at clonal density and the numbers of neurospheres were counted after 1 week.

### ELISA

Enzyme-linked immunosorbent assays (ELISAs) were purchased from R&D Systems for CD40 (Catalog #MCCD40) and used for the analysis of microglia-derived CM samples as per the manufacturer's instructions for cell culture supernatant samples.

### qPCR

Spinal cord and brain neurospheres were collected in Buffer RLT (QIAGEN, Catalog #: 79216) with b-mercaptoethanol. Samples were processed according to the manufacturer’s directions using the RNeasy Micro Kit (QIAGEN, Catalog #: 74004), including treatment with the RNase-free DNase Set (QIAGEN, Catalog #: 79254). cDNA synthesis was carried out with Superscript III First Strand Synthesis System (Invitrogen, Catalog #: 18080051). qPCR was performed on a 7900HT Fast Real-Time PCR System (Applied Biosystems, Catalog #: 4329001). Cycling conditions consisted of initial activation (2 minutes at 50 °C and then 10 minutes at 95 °C), followed by 40 cycles of 15 seconds at 95 °C and 1 minute at 60 °C, followed by 15 seconds at 95 °C, 15 seconds at 60 °C, and 15 seconds at 95 °C. Primers were obtained from Bio-Rad Laboratories (Canada, Cat #: qMmuCED0040561 (CD40), qMmuCID0023528 (CD40L), and 10025636 (GAPDH).

### Statistics

Data are represented as means ± SEM. Two‐tailed *t*-tests were performed to compare between 2 groups. One‐way ANOVAs were used to compare multiple groups with Dunnett’s post hoc test. Two‐way ANOVAs were used to compare multiple groups with Tukey’s post hoc test. Significance is considered *P* < .05. All graphs and analyses are generated from Excel (Microsoft) or GraphPad Prism 9 (Graph Pad Software).

## Results

### Spinal cord niche cells release a 10-30 kDa inhibitory factor in response to MBP exposure

**Figure 1.**
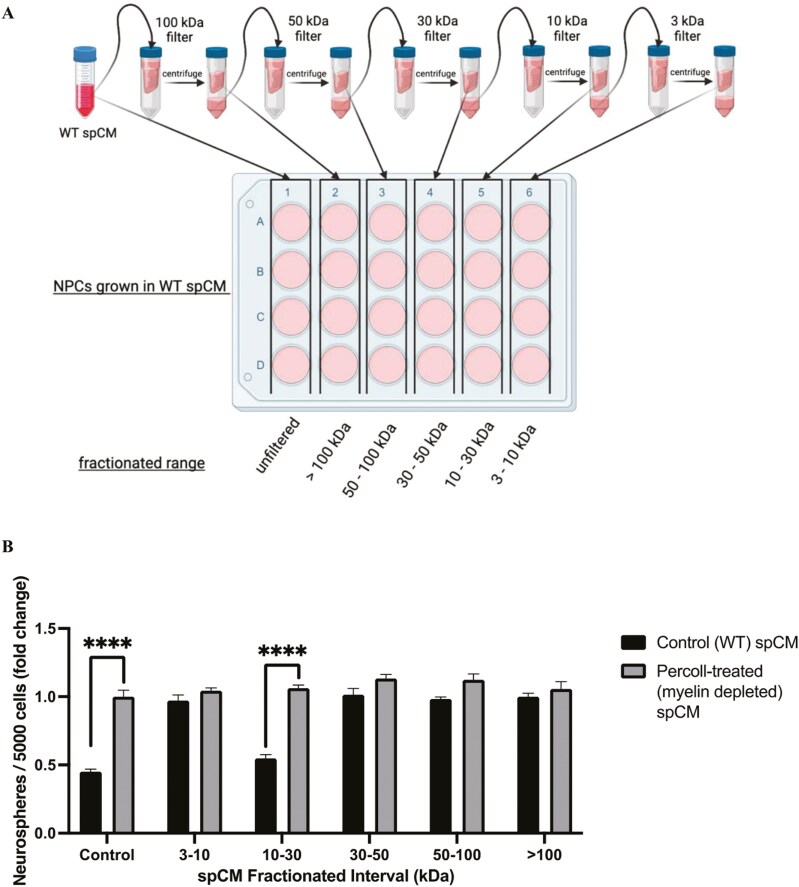
The molecular weight of the inhibitory factor is 10-30 kDa. (A) Experimental paradigm for CM fractionation. (B) There is a significant reduction in neurospheres formation in the 10*-*30 kDa molecular weight fraction from WT, but not myelin-depleted, CM (*n* = 4 independent experiments). Control cultures were grown in EFH media alone. Data represents % fold change ±SEM. ****P < .0001.

Previous work has shown that a heat-labile factor is present in spinal cord conditioned medial (spCM) exposed to MBP that inhibits the proliferation of NSCs, without affecting their survival, as measured by a reduction in the numbers of NSC-derived colonies (termed neurospheres) in vitro.^19,22^ In the first series of experiments aimed at characterizing the released factor, we performed a size-exclusion centrifugal filtration assay on spCM. spCM from WT cultures (which contain MBP) and following MBP exclusion using Percoll was fractionated based on molecular weight (>3 and <10 kDa, >10 and <30 kDa, >30 and <50 kDa, >50 and <100 kDa, and >100 kDa) using sequential centrifugal filtration (Figure 1A). We asked which of these fractions contained the inhibitory factor by growing passaged neurosphere-derived cells in each of the spCM fractions (Figure 1B). The numbers of neurospheres were assessed in unfractionated spCM (control) and fractionated spCM. Consistent with previous reports,^24^ spCM that was exposed to MBP resulted in a significant 55 ± 8% reduction in neurosphere formation compared to spCM that was produced in the absence of MBP (*P* < .0001). Interestingly, this same inhibitory effect was only detected when spNSCs were grown in the 10-30 kDa fraction (48 ± 5% reduction in neurospheres in MBP containing spCM, *P* < .001). Hence, the inhibitory factor is in the 10-30 kDa molecular weight range.

### Microglia are the source of the inhibitory factor

**Figure 2.**
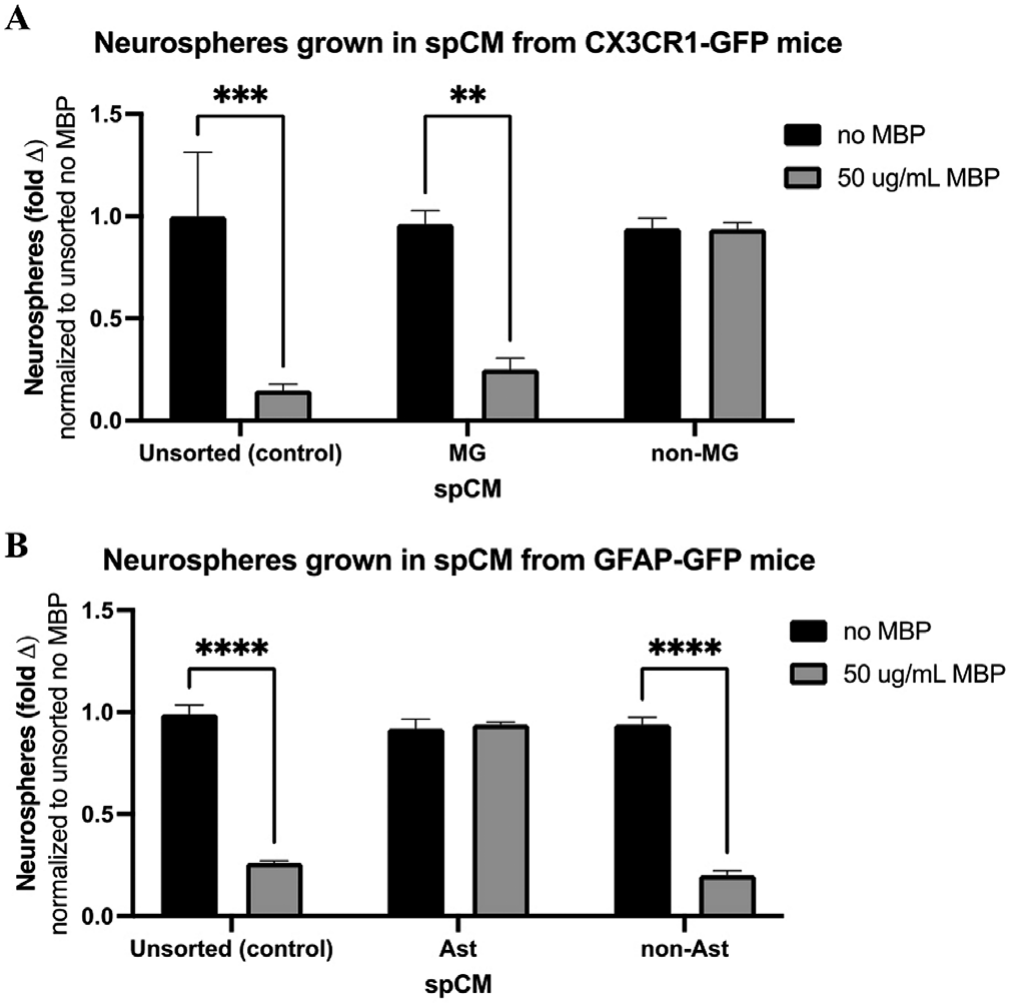
Microglia from the spinal cord PVZ are the cell source for the inhibitory factor. (A) Microglia spCM (MG-spCM) grown in the presence of MBP is inhibitory to neurosphere formation. Primary neurosphere cultures grown in spCM from GFP+ microglia (from CX3CR1-GFP mice) were collected following exposure to exogenous MBP (50 μg/mL, gray bar) or in the absence of MBP (black bar). MG, microglia sorted fraction (GFP+) and non-MG, microglia-negative (GFP−) (*n* = 4 independent experiments; **P < .01, ***P < .001) (B) CM from spinal cord astrocyte cultures has no effect on neurosphere formation. Primary neurosphere cultures were grown in spCM from GFAP-GFP mice grown in the presence (gray bar) or absence (black bar) of MBP (50 μg/mL). Ast, astrocyte sorted fraction (GFP+) and non-Ast, astrocyte-negative (GFP) (*n* = 4 independent experiments; ****P < .0001).

To determine which spinal cord niche cell type was the source of the inhibitory factor, we performed fluorescence-activated cell sorting (FACS) from transgenic mice that have green fluorescent protein (GFP) expressing microglia (CX3CR1-GFP mice) or astrocytes (GFAP-GFP mice). The PVZ from the spinal cords of CX3CR1-GFP or GFAP-GFP mice were microdissected and sorted for GFP-expressing cells (Supplementary Figure S1A and B). Unsorted (control), GFP+, and GFP− cells were plated in the presence or absence of recombinant MBP (50 μg/mL) to generate spCM.^22^ This was done to reintroduce MBP to the cells as myelin was removed during pre-sort tissue processing to reduce debris particles. As shown in Figure 2A, spCM collected from the microglia cultures grown in the presence of MBP had significantly reduced neurosphere numbers compared to that grown in the absence of MBP (74 ± 17% reduction in neurosphere numbers, *P* = .005). Notably, spCM from the microglial-negative fraction (GFP− from CX3CR1-GFP mice, non-microglia) was not inhibitory to neurosphere formation.

We performed the same experiment using spCM collected from astrocyte cultures (GFP+ from GFAP-GFP mice, astrocyte-spCM). As shown in Figure 2B, astrocyte-spCM did not inhibit neurosphere formation. Notably, the spCM derived from the GFP-fraction of GFAP-GFP sorted cells, which does contain microglia, was also inhibitory to neurosphere formation when exposed to MBP (79 ± 9% reduction in neurosphere number; *P* < .0001). Taken together, these findings reveal that microglia are the cells that release a 10-30 kDa factor that inhibits NSC proliferation.

### Soluble CD40 released from microglia is inhibitory to neurosphere formation

**Figure 3.**
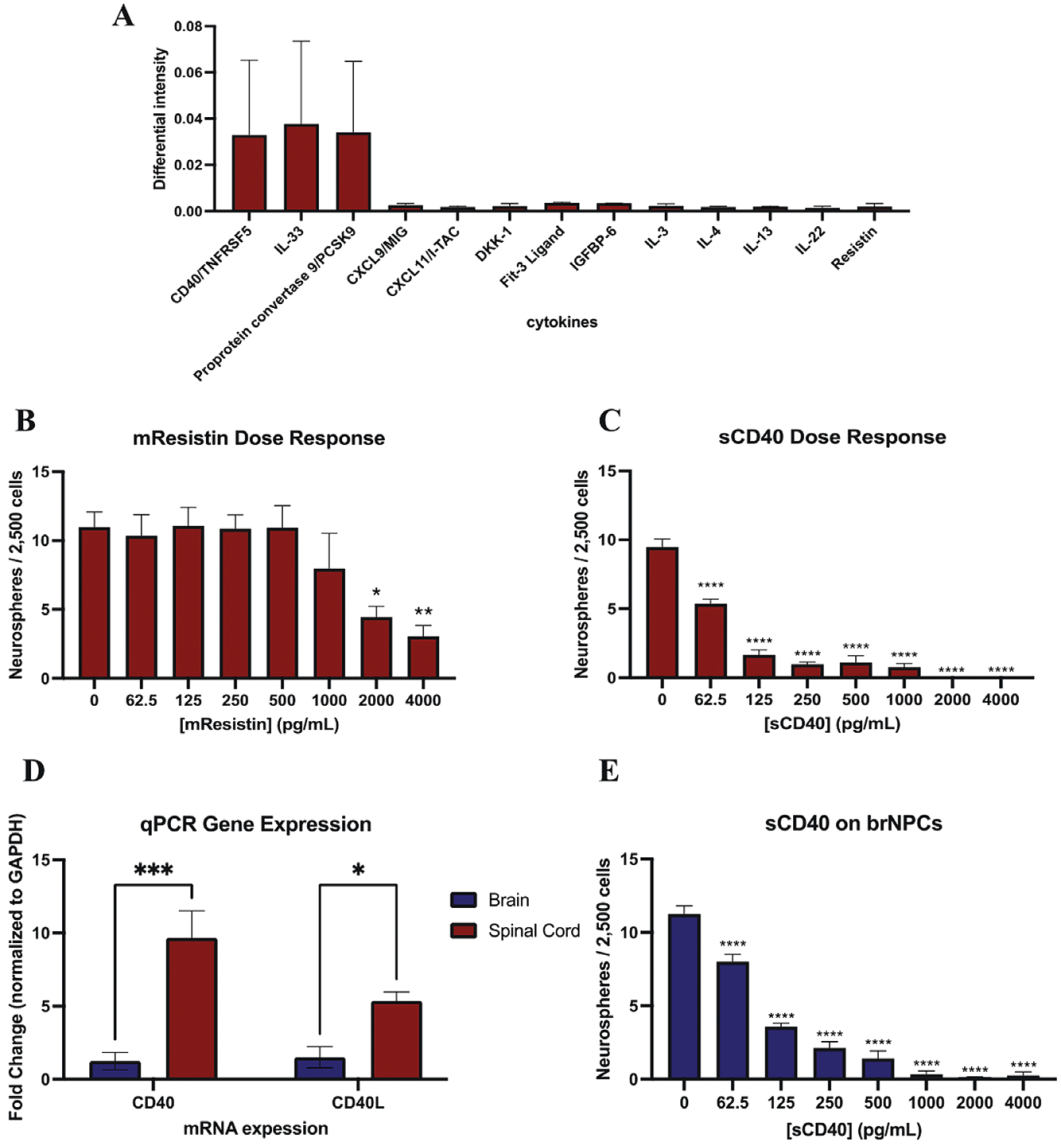
Soluble CD40 is the microglia-derived inhibitory factor. (A) Cytokine array analysis reveals 13 factors in the 10-30 kDa molecular weight range that are more highly expressed in MG-spCM with MBP compared to MG*-*spCM grown in the absence of MBP. The differential intensity was determined by subtracting cytokine intensities of MG-spCM with MBP from MG-spCM minus MBP. (*n* = 3 independent experiments) (B) Dose response of mResistin shows inhibition of neurosphere formation at 2000 and 4000 pg/mL (*n* = 4 independent experiments, *P < .05, **P < .01) (C) Dose response of sCD40 shows an inhibitory effect on neurosphere formation at all concentration examined from the spinal cord (*n* = 4 independent experiments, ****P < .001). (D) qPCR of CD40 receptor (CD40) and ligand (CD40L) expression in brain and spinal cord derived neurospheres (*n* = 3 independent experiments, ***P < .001, *P < .05). (E) Dose response of sCD40 on brain neurosphere formation (*n* = 4 independent experiments, ****P < .0001).

We next performed a cytokine array on spinal cord microglia-CM (microglia-spCM) derived from GFP+ cells sorted from CX3CR1-GFP transgenic mice, with and without recombinant MBP and compared the expression of factors between the 2 microglia-spCM conditions. The cytokine array revealed greater expression of 26 factors in MBP-exposed microglia-spCM compared to microglia-spCM grown in the absence of MBP (Supplementary Figure S2). Of these, 13 were 10-30 kDa in size (Figure 3A). We focused our attention on 2 factors, resistin and soluble CD40 (sCD40), as both have been shown to regulate the proliferation kinetics of various cell populations. Resistin is a 12.5 kDa protein that enhances the proliferation of mesenchymal, smooth muscle, and granulosa cells.^26-29^ CD40 plays a role in microglia, B cell, and cancer cell proliferation.^13-15^ CD40 is a 48kDa transmembrane protein that can undergo enzymatic cleavage (proteolysis) of its extracellular domain which releases a soluble extracellular component of the protein (sCD40) that is 18 kDa in size.^16^

We asked whether our candidate molecules regulated NSC behavior by performing dose-response curves with mouse resistin (mResistin) or soluble CD40 (sCD40) (0-4000 pg/mL concentrations) using the neurosphere assay. mResistin was inhibitory to neurosphere formation at concentrations in the ng/mL range (59 ± 19% and 72 ± 20% reduction of neurosphere formation at 2000 pg/mL (*P* = .029) and 4000 pg/mL (*P* = .0072) concentrations of mResistin, respectively) (Figure 3B). sCD40 significantly inhibited neurosphere formation in the pg/mL range (62.5 pg/mL; 43 ± 10% reduction in the numbers of neurospheres; *P* < .0001) (Figure 3C). Due to the order of magnitude difference in sCD40 compared to mResistin concentration in the cytokine array, we performed an ELISA to determine the concentration of sCD40 in microglia-spCM and assess whether sCD40 could account for the CM-mediated inhibition (Supplementary Figure S3A**).** The concentration of sCD40 was significantly greater in microglia-spCM +MBP compared to microglia-spCM without MBP (73.6 ± 17.8 pg/mL vs 1.2 ± 0.3 pg/mL; *P* = .0216) and all microglia-negative spCM had negligible sCD40 expression (1.16 ± 0.01 pg/mL and 0.980 ± 0.084 pg/mL, with and without MBP, respectively; *P* > .9999). Thus, the concentration of sCD40 in microglia-spCM grown in the presence of MBP was consistent with the dose-response curve, revealing significant inhibition of neurosphere formation in the 62.5-125 pg/mL range and supporting the hypothesis that sCD40 is the inhibitory factor released from microglia in the presence of MBP.

### Neural precursor cells from the brain and spinal cord express CD40/TNFRSF5 and CD40L

We predicted that brain and spinal cord NPCs would express CD40 receptor and CD40L based on previous findings that spCM is inhibitory to neurosphere formation from both regions.^22^ Using qPCR we found that both brain and spinal cord-derived neurospheres expressed CD40 receptor and CD40L, with significantly greater expression of CD40 receptor and CD40L in spinal cord NPCs compared to brain NPCs (8.7 ± 3.4-fold increase in CD40 receptor expression in spinal cord vs brain NPCs, *P* = .0006; 3.55 ± 3.51-fold increase in CD40L expression in spinal cord vs brain NPCs, *P* = 0.0359) (Figure 3D). Consistent with this observation, brain neurospheres were significantly reduced in the presence of sCD40 (29 ± 9% reduction at 62.5 pg/mL; *P* < .0001) (Figure 3E). Thus, brain and spinal cord NPCs can respond to sCD40 released from spinal cord microglia.

## Discussion

Herein, we have shown that spinal cord microglia, in the presence of MBP, release sCD40 which can interact with the membrane-bound CD40 receptor pathway found on forebrain and spinal cord neural precursor cells to inhibit their proliferation. CD40 is a transmembrane protein that acts as a receptor for CD40 ligand (CD40L). The binding of CD40 to CD40L has been shown to induce proliferation in a variety of cell types.^15^ In microglia, proteolysis of CD40 or direct expression of sCD40 mRNA isoform releases sCD40 (which is ~18-21 kDa in size) and binds/sequesters extracellular CD40L.^17^ This prevents the binding of membrane-bound CD40 receptor to its target (CD40L) and leads to inhibition of proliferation in CD40 receptor-expressing NPCs. Previous groups have demonstrated expression of CD40 in NSCs,^30^ consistent with our findings herein. Our findings that CD40 and CD40L are also expressed in neurosphere-derived cells support a mechanism of action whereby sCD40 reduces CD40-CD40L interactions on NSCs by blocking the active site of CD40L.

Microglia are known to express CD40 on their membrane.^31^ Other cell types that express CD40 release sCD40 through the proteolysis of the extracellular domain,^32^ however, this mechanism has not been demonstrated in microglia. Indeed, there is evidence suggesting that CNS cells such as microglia and astrocytes may express sCD40 through alternative splicing specific for the exons that code extracellular domain portions of CD40.^33^ Given that MBP is a highly positively charged protein, it is possible for MBP to interact with cells through electrostatic interactions with the negatively charged lipid bilayer of the cell membrane.^34-37^ Indeed, MBP has been shown to interact and take part in lipid raft formation intracellularly and thus could act as a nucleation point for lipid rafts in the extracellular domain as well.^38,39^ In previous studies^21^ we have shown that the large positive charge of MBP is necessary for its inhibitory effect on NSCs. This could, in theory, be sufficient to activate proteolysis of membrane CD40 or transduce an intracellular signaling cascade to express sCD40 through alternative splicing. Further investigation is needed to determine which of these mechanisms is responsible for the production of sCD40 in response to MBP exposure. This is important as factors that prevent this proteolysis or alternative splicing from occurring are potential therapeutic targets to increase NPC proliferation following injury and support NPC-mediated neural repair.

Importantly, previous reports from our lab have demonstrated that primary spinal cord CM (which contains sCD40 from microglia that were exposed to MBP) is inhibitory to proliferation in vivo.^22^ Moreover, the exposure to MBP temporarily inhibits proliferation but does not lead to cell death as was revealed by the return of neurosphere formation following MBP removal.^19^

We have also shown that the inhibitory factor is a heat-labile factor, which we have now identified as microglia-derived sCD40.^22^ The structure of sCD40 is consistent with it being a heat-labile structure based on the cysteine-rich domain that has an unusually positioned disulfide bridge that allows the ladder-like structure of sCD40.^40^ The breaking of the bonds occurs with exposure to extreme heat (as is done in heat-inactivation) which prevents the refolding of this protein due to the unusual tertiary protein structure^41^ and thus is consistent with our previous findings.

Our work has highlighted the cell-based mechanism that underlies the MBP-mediated inhibitory effects on NSC proliferation. Based on the literature, the signaling pathways that could potentially mediate this inhibition involve the recruitment of tumor necrosis factor-associated factors (TRAFs) on the cytoplasmic component of CD40 which clusters on the cell membrane.^40^ Depending on which of the six different TRAF proteins are recruited, there are different downstream intracellular signaling pathways that can be initiated—this can range from the canonical and/or non-canonical nuclear factor κB (NFκB)-signaling pathways, mitogen-activated protein kinases (MAPKs), phosphoinositide 3-kinase (PI3K), and/or phospholipase Cγ (PLCγ) pathway.^40^ Specifically, recent evidence has shown that TRAF5 has a proliferative effect on B cells and tumor cells through NFκB and MAPK intracellular signaling pathways^41-43^ making these pathways potential candidates for the proliferative regulation of MBP on NSCs.

The finding that spinal cord microglia interact with MBP to inhibit NSC proliferation yet MBP presented to forebrain niche cells does not regulate cell proliferation^22^ speaks to the spatial heterogeneity of microglia along the neuroaxis.^44,45^ Microglia heterogeneity is an area of much interest and recent studies have noted significant microglia heterogeneity both regionally and temporally throughout the CNS.^44,46–48^ Our findings suggest that one potential difference could be the composition of the cell membrane. For example, spinal cord PVZ microglia may have more negatively charged phospholipids or different lipid-raft receptor complexes that interact with extracellular MBP compared to forebrain SVZ microglia. Notably, our experimental design rationale was to include PVZ microglia that were in close proximity to the NSC niche (within or spatially restricted to the gray matter). Hence, while we have shown that PVZ spinal cord microglia respond to MBP we cannot rule out the presence of gray matter microglia surrounding the central canal. Further, since we specifically removed the white matter from our dissections, it is possible that white matter-derived microglia play a similar role in regulating NSC activity. Examining the impact of spatially distinct microglia pools would be an interesting next step and potentially reveal further microglia heterogeneity within the spinal cord. An understanding of microglia heterogeneity could also provide insight into a therapeutic target whereby the MBP interaction with spinal cord microglia would be blocked which could potentially enhance NPC-mediated neural repair.

## Conclusion

Together, this work has elucidated the cellular and molecular signals that mediate the inhibitory effect of MBP on NSCs proliferation in the spinal cord. Identification of the mechanism of action will aid in how NPC-based therapies for spinal cord injury (SCI) repair are approached since significant amounts of extracellular MBP can be found in patients for months after an initial CNS injury.^49,50^ Thus, therapeutic interventions utilizing endogenous or exogenous NPC populations would benefit from considering the microglia-mediated sCD40 effects on NPCs.

## Supplementary Material

sxae076_suppl_Supplementary_Figures

## Data Availability

The data that support the findings of this study are available on request from the corresponding author.
