# Supplementary material for: Microglia in the spinal cord stem cell niche regulate neural precursor cell proliferation via soluble CD40 in response to myelin basic protein
Source: Stem Cells. 2024 Nov 16;43(2):sxae076. doi: 10.1093/stmcls/sxae076 (PMC11878629; doi:10.1093/stmcls/sxae076)
Supplement: sxae076_suppl_Supplementary_Figures [file sxae076_suppl_supplementary_figures.pdf]

SUPPLEMENTAL FIGURES

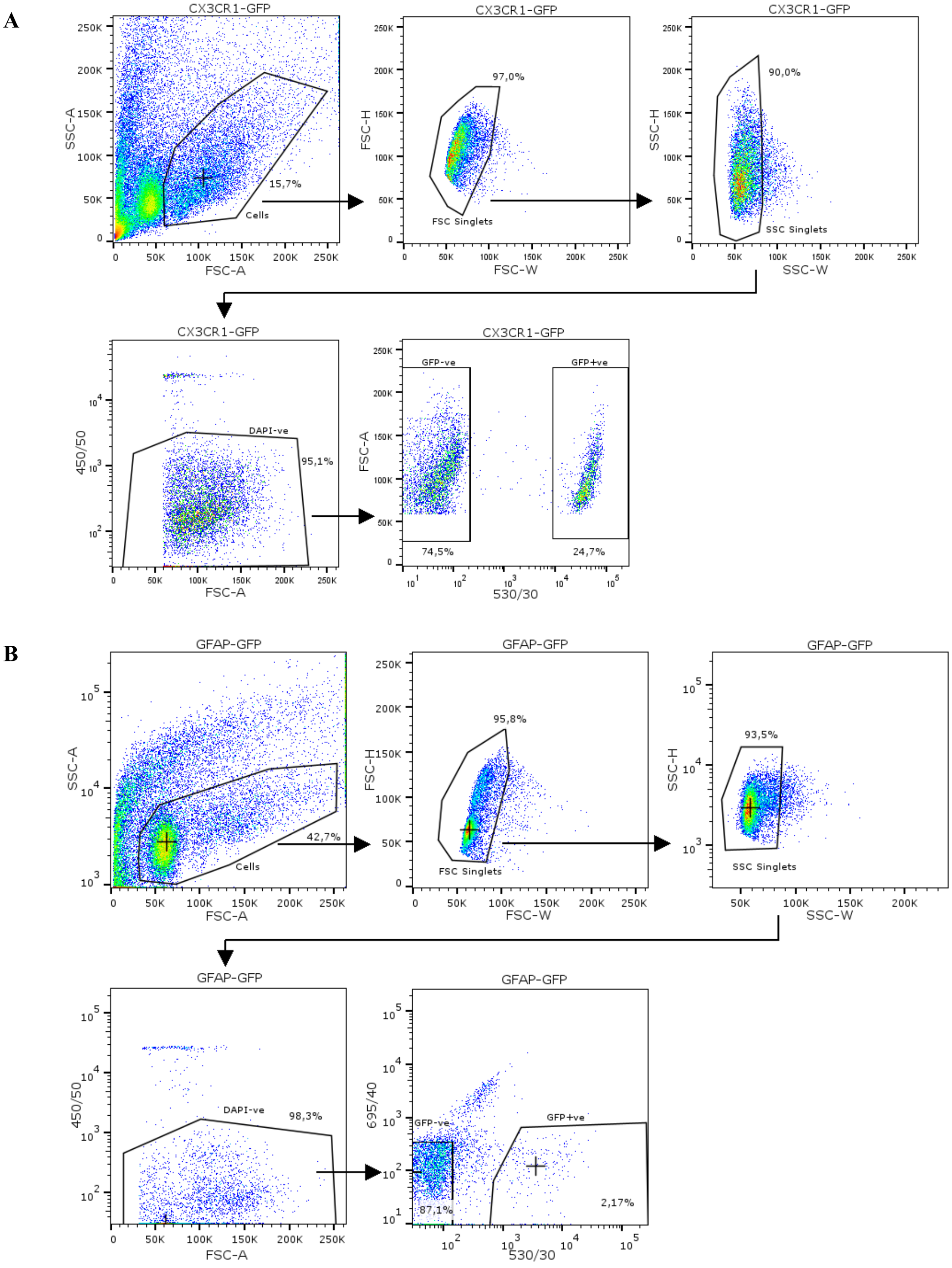

Figure S1. Representative FACS scatterplots demonstrating the gating strategies used to isolate GFP+ and GFP- cells from (A) CX3CR1-GFP mice, where GFP+ cells are microglia, and from (B) GFAP-GFP mice, where GFP+ cells are astrocytes. Gate edges were determined using appropriate negative (i.e. DAPI-, GFP-) controls.

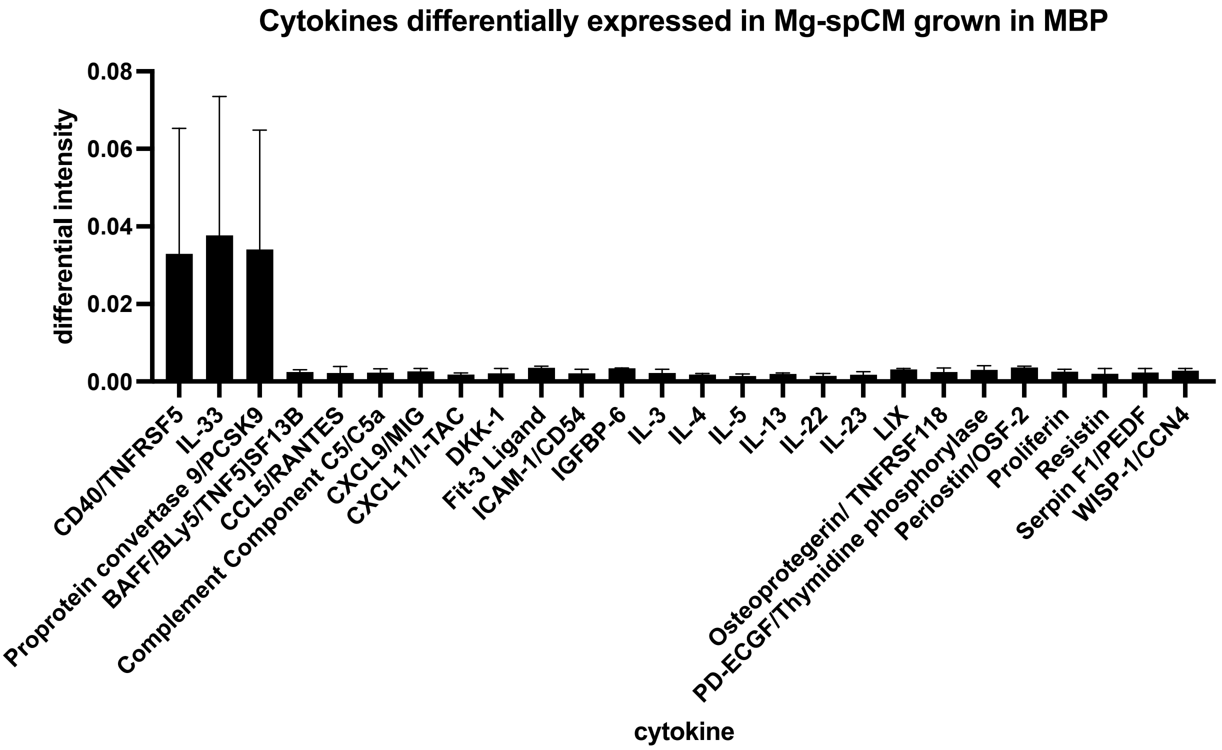

Figure S2. Bar plot of 26 cytokines that are more highly expressed in MG-spCM with MBP compared to MG-spCM without MBP.

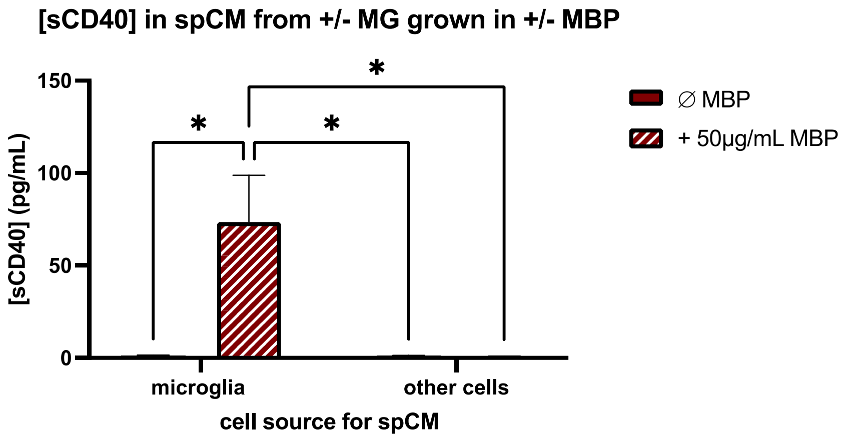

Figure S3. Bar plot of sCD40 concentrations from MG-spCM grown in the presence (50ug/mL) or absence of MBP.
